# Investigating time-dependent COVID-19 pandemic mental health data: Challenges and opportunities of using panel data analysis

**DOI:** 10.1371/journal.pmed.1004219

**Published:** 2023-04-18

**Authors:** Mariana Pinto da Costa, Robert Stewart

**Affiliations:** 1 Institute of Psychiatry, Psychology & Neuroscience, King’s College London, London, United Kingdom; 2 South London and Maudsley NHS Foundation Trust, London, United Kingdom

## Abstract

Mariana Pinto da Costa and Robert Stewart provide commentary on a large prospective panel survey of mental health during the pandemic and consider the implications of such data science initiatives.

Amidst the widespread ramifications of the Coronavirus Disease 2019 (COVID-19) pandemic, its impact on population mental health has attracted considerable interest [1], with the potential for adverse mental health consequences widely recognised at an early stage. The spring of 2020 saw a proliferation of online surveys, many focusing on mental health and well-being [2], but unlikely to provide robust research output because of design limitations. However, among these numerous initiatives, some important and more carefully considered projects were set up such as the large, prospective panel survey reported by Feifei and colleagues in PLOS Medicine [3]. This follow-up of an online sample of over 57,000 adults in England from March 2020 to April 2022 was able to evaluate associations of depressive/anxiety symptoms with both contextual factors (the “stringency” of lockdown policy and COVID-19 deaths) as well as with individual factors (such as confidence in institutions, concerns about COVID-19, and social support). Particular advantages lie in the large size of the sample, the relatively high follow-up rates, and frequently repeated measures over the time period of interest. Of course, not everyone has access to or interest in online surveys and, although steps were taken with oversampling and weighting techniques to improve representation, the people who participated in the study are unlikely to be nationally representative, as acknowledged by the authors.

Within these limitations, the study does provide a good opportunity to measure population-level changes in reported symptoms of depression and anxiety over the course of the pandemic and to investigate the factors influencing these. In particular (and perhaps rather bravely, given continuing acrimonious social media debates), the authors attempt to disentangle the causal impact of the pandemic from that of the policies taken to reduce viral transmission. There are self-evident challenges in this exercise with so many events occurring over the same time periods—the direct and indirect consequences of infections, the direct and indirect consequences of “lockdown” and other policies, co-occurring changes in the national economic outlook, changes in healthcare provision and access. Although factors such as isolation, financial instability, and uncertainty about the future are obvious potential explanations for the upswing in symptoms of depression and anxiety observed during the pandemic [4], they are likely to depend substantially on individuals’ personal circumstances. For example, for one person, a working-from-home policy might provide relief from social stressors and improve work-life balance, saving commute travel time and costs; for another, this may result in stressful family relationships, challenging childcare, or loneliness. Adolescents and young adults appear to have been particularly vulnerable to early-pandemic adverse mental health [5], but continued fears about infection and other health threats, as well as self-imposed isolation, may translate into a higher mental health burden for older adults in years to come. No attempt was made in the analysis by Bu and colleagues to investigate this sort of variation in effect, although presumably it will be a focus for further work with these data.

Although large datasets, like the cohort reported by Bu and colleagues, can be helpfully informative, interpretation can also be complicated. Very large samples can identify associations at high levels of statistical significance which are not necessarily clinically important—for example, although relationships are reported here between policy and depressive/anxiety symptoms, most of the changes are very subtle (e.g., a 0.23 increase on a 27-point depressive symptom scale, for each standard deviation increment in policy “stringency index”). Furthermore, it remains impossible to conclude whether associated factors are risk factors or risk markers. Thus, the marked decline in symptoms during the first lockdown, followed by a steady increase to early 2021, tracks changes in policy more closely than changes in COVID-19 case numbers; however, it could have nothing to do with policy changes and might instead reflect a collective realisation that the pandemic was not going to resolve soon, perhaps also explaining the rise (in depressive symptoms at least) into the winter of 2021/2022 regardless of policy changes. In the end, as the authors acknowledge, most of these associations are weak, and there is a risk of overinterpreting them against much stronger and long-recognised risk factors for mental distress. In addition, bidirectional influences cannot be ruled out.

It is also important to bear in mind that mean symptoms levels of depression/anxiety from brief screening scales do not necessarily equate to occurrence of clinical disorders. A “dimension-disorder” relationship has been proposed in other health specialties, such as that between population-level blood pressure and prevalence of hypertension [6]; however, a comparable relationship between population-level well-being and mental disorder prevalence (or severity) is unproven at best [7,8]. For people with a preexisting mental disorder, a period of shared national hardship may be easier, or at least less isolating, than times when everyone else seems to be in happier circumstances, so paradoxical patterns of risk are possible, at least in theory. Further evidence is needed on prevalence and outcomes of diagnosable mental disorders, particularly given the profound changes in mental health service provision that have occurred as a result of the COVID-19 pandemic [9–11], not to mention the disruption to wider healthcare provision on the background of sizeable preexisting health inequalities.

Clearly, the study reported here focuses on lessons that might be learned for future pandemics (e.g., to mitigate adverse mental health outcomes), albeit dependent on the extent to which recent experiences generalise. However, perhaps more importantly it demonstrates a data science initiative with both immediate and longer-term implications, namely the potential for scaled-up real-time surveillance of mental health trends. There are likely to be continuing population-level mental health impacts of the pandemic, as well as challenges arising from other world events, economic stressors, and compromised healthcare provision; however, until recently, there have been very few adequate resources to inform mental health policy compared, for example, to those monitoring cancer and cardiovascular disease. “Public mental health” at times can seem a little distant as an aspiration, but pandemic-accelerated capabilities such as well-designed, rapidly implemented panel surveys are likely to be an important step in the right direction.
